# Editorial: Sonification, aesthetic representation of physical quantities

**DOI:** 10.3389/fnins.2023.1162383

**Published:** 2023-03-17

**Authors:** Diego Minciacchi, Riccardo Bravi, David Rosenboom

**Affiliations:** ^1^Physiological Sciences Section, Department of Experimental and Clinical Medicine, University of Florence, Florence, Italy; ^2^The Herb Alpert School of Music, California Institute of the Arts, Valencia, CA, United States

**Keywords:** auditory representation, music, aesthetics perception, information processing, brain computer interface, data navigation

The practice of turning scientific data into structured audio, also known as sonification, is nowadays reaching targets never thought possible just a decade ago. In the last years there was an expansion of the employment of sonification techniques to various scenarios highlighting the potential of auditory representation for data exploration (Minciacchi and Rosenboom, 2020). Driven by analogies between the structure of proteins and many forms of music, attempts to develop sonification algorithms for representing protein sequence data using sound have been implemented (Martin et al., 2021). Additionally, using deep learning models, researchers generated *de novo* musical scores and translated pitch information and chain lengths into sequences of amino acids to design *de novo* proteins (Yu and Buehler, 2020). The potential for this to play an important role as an auxiliary tool for medical surveillance has also been explored (e.g., suspicion of myocardial infarction: Aldana Blanco et al., 2022). Besides, movement sonification was shown to provide a significant connection between movement and cognition, which fosters the design of learning environments that promote creativity (Oppici et al., 2020). To achieve accurate auditory representation of data, many factors are to be considered when designing sonification algorithms. Most are related to the type of data and the choice of parameters that would best represent and its significance. The integrative power of auditory perception can hence facilitate the discovery of hidden order or patterns in data and parsing subtle differences in types of complexity.

The articles presented in this Research Topic offer a concentrated and updated perspective of methods and results available nowadays in sonification. Kantan et al. described an interactive system for the sonification of biomechanical quantities. A sit-to-stand sonification model for rehabilitative and monitoring applications was designed and developed. The model, based on the integration of rhythmic and harmony-based elements, has been shown to provide a conceptual and technical starting point in the quest to leverage the potential of the sonic medium in rehabilitative applications. A closer relationship to the orthodox meaning of music is the contribution by Sköld and Bresin in which the sonification of complex spectral structures as a notation system for representing sound-based musical structures was presented as well as the design of a system for visual representation and sonification of sound notation. The development of automatic analysis of sound structures to provide transcriptions of sound-based music in a software environment was also proposed for future work. Moreover, the study by Riley examined whether auditory-assisted sonification feedback would enhance music students' perception of the interpretive nuances and could be used as a tool to magnify interpretation and improve performance. Authors showed that—in the context of music performance—auditory-assisted sonification triggered more intensive listening and increased the coupling between visual and auditory perception and motor production. Furthermore, the contribution by Panariello and Bresin examined models of sonification for personal computer shutdown and idle mode processes using two aesthetically complex sound models. Their approach and encouraging results suggest that the sonification of computer processes and software functioning could be the basis for the creation of aesthetically complex soundscapes connecting the user to the essential activities of digital devices. Finally, Ziemer and Schultheis introduced a method to supplement sonification designs and evaluation methods from a perceptual viewpoint. The approach is based on the extensive consensus that psychoacoustics plays a significant role in data sonification. An experimental approach centered on noticeable differences using the maximum likelihood procedure is described. The method is implemented to serve as a ground for the perceptual linearity of the sonification mapping process and to recognize perceptual properties such as resolution, hysteresis effects, and perceptual interferences during the sonification evaluation stage. The choice of sonification design and data supervising appear to be hot subjects substantiated in this topic and more recent literature. As a final example, it is worth mentioning experiences of movement sonification in sports-related performances, enabling new insights into the relationships between auditory perception and the neuromuscular system (O'Brien et al., 2020). It seems definitively plausible to affirm that the large potential of sonification strategies is starting to be exploited.

## Author contributions

Conception, writing, editing, and approving: DM, RB, and DR. All authors contributed to the article and approved the submitted version.
